# Impact of Provider Volume on Intraprocedural Rupture Risk During Endovascular Treatment of Ruptured Intracranial Aneurysms: A Systematic Review and Meta-Analysis

**DOI:** 10.3390/life16091393

**Published:** 2026-08-24

**Authors:** Chao-Ya Yang, Kuo-Wei Chen, Fon-Yih Tsuang, Furen Xiao, Chung Liang Chai

**Affiliations:** 1 Neurosurgery Department, National Taiwan University Hospital, Taipei 100229, Taiwan; vivianyang03@gmail.com (C.-Y.Y.); tsuangfy@gmail.com (F.-Y.T.); xfr@dr.com (F.X.); 2 Neurosurgery Department, National Taiwan University Hospital Hsin-Chu Branch, Hsinchu 300195, Taiwan; duckgoway@gmail.com; 3 Neurosurgery Department, Yee Zen General Hospital, Taoyuan 32645, Taiwan

**Keywords:** intraprocedural rupture, endovascular coiling, ruptured intracranial aneurysm, provider volume, aneurysm location, systematic review

## Abstract

Intraprocedural rupture (IPR) during coiling of ruptured intracranial aneurysms is a serious complication. In this study, we assessed whether center volume influences the risk of IPR. For this purpose, we conducted a systematic review (1996–present) including a final total of 98 studies. Centers were classified as high-volume (≥20 cases/year) or low-volume (<20), with the IPR rate as the primary outcome, and subgroup analyses by vascular territory and meta-regression were performed to quantify volume effects. The IPR rate was lower at high-volume centers than low-volume centers (3.5% [95% CI, 3.01–4.06] vs. 5.37% [95% CI, 4.06–7.07]; *p* < 0.01). Subgroup analyses by territory favored high-volume centers for anterior cerebral artery aneurysms (5.41% vs. 17.24%; *p* = 0.01) and middle cerebral artery aneurysms (7.49% vs. 50.0%; *p* < 0.01), while posterior circulation showed no significant difference (8.76% vs. 3.61%; *p* = 0.14). The meta-regression showed a statistically significant association in the overall IPR rate, with a regression coefficient of −0.65 (*p* = 0.0047). These findings potentially suggest a volume–outcome relationship, in which a higher annual case volume is associated with a lower risk of IPR during coiling of ruptured intracranial aneurysms.

## 1. Introduction

Intraprocedural rupture (IPR) during endovascular coiling of ruptured cerebral aneurysms is a rare but devastating event, with reported rates in the range of 1–10% and mortality reaching up to 40% [1]. As IPR worsens neurological outcomes and prolongs hospitalization, identifying modifiable determinants is clinically important. Across endovascular disciplines, higher hospital and operator volumes are linked to better outcomes, including fewer perioperative complications after endovascular aortic aneurysm repair and peripheral interventions [2,3,4]; lower mortality for ruptured abdominal aortic aneurysms treated endovascularly [5]; improved reperfusion and functional outcomes after thrombectomy [6]; and reduced periprocedural ischemic events with higher annual carotid stenting volumes [7]. In a study focused on aneurysmal subarachnoid hemorrhage care, higher-volume centers showed lower in-hospital mortality after clipping or coiling, although IPR-specific effects were not assessed [8]. Known patient, aneurysm, and technical predictors—such as small size, irregular morphology, wide neck, and catheter instability—do not fully explain institutional variability, suggesting that experience, workflow, and multidisciplinary coordination play non-negligible roles in this context [9,10,11]. A key controversy is whether volume per se reduces IPR risk across vascular territories, or whether the case mix and anatomy dominate outcomes. This study systematically evaluates the relationship between hospital provider volume and the IPR rate during coiling of ruptured aneurysms by comparing high-volume (defined as ≥20 cases/year, based on the Brain Attack Coalition consensus recommendations for Comprehensive Stroke Center certification [12,13]) with low-volume centers, with the aim of determining whether higher annual provider volume is associated with a lower IPR rate. Our principal conclusion is that higher-volume centers have lower IPR risk, supporting volume-based referral and system-level organization to enhance procedural safety.

## 2. Materials and Methods

Study design and registration: We conducted a PRISMA-compliant single-arm systematic review and meta-analysis focused on IPR during endovascular coiling for ruptured intracranial aneurysms, which was prospectively registered in PROSPERO (CRD42023417383; 11 October 2023). All data and materials are provided within the article and are freely available via the Open Science Framework (OSF); repository details and access links will be provided during review and available prior to publication.

**Figure 1:**
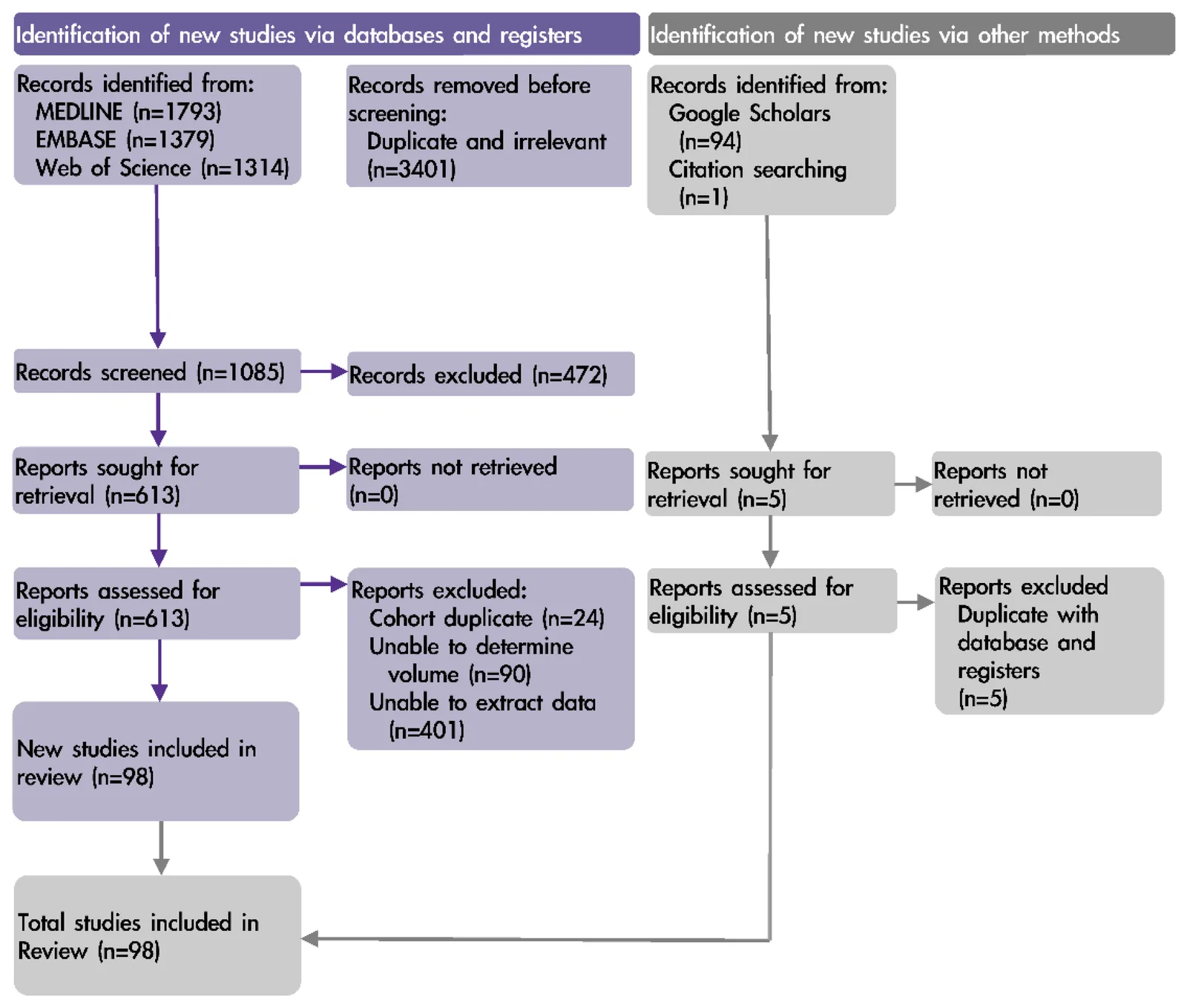
PRISMA flow diagram of study enrollment and selection. The diagram illustrates the identification, screening, eligibility assessment, and inclusion of studies in the systematic review and meta-analysis.

Data sources and search strategy: We searched MEDLINE, Embase, Web of Science, and Google Scholar on 10 September 2023, without language restrictions. The full database search strategies are detailed in Supplementary Material S1, and the PRISMA flow diagram is shown in Figure 1.

Eligibility criteria: We included clinical studies (randomized or non-randomized) reporting IPR among patients with ruptured intracranial aneurysms treated via endovascular coiling. We excluded unruptured aneurysms and incompatible pathologies (e.g., arteriovenous malformation, dissection/dissecting aneurysm, blood-blister-like aneurysm), as prespecified in the protocol (PROSPERO). Flow-diverting stents and intrasaccular flow disruption were also excluded.

Study selection and data extraction: Two reviewers (C.C. and K.C.) independently screened titles/abstracts and full texts using a pre-piloted form (Supplementary Material S2), and disagreements were resolved with a third reviewer (F.T.). HubMeta was used to de-duplicate and filter irrelevant records. From each study, we extracted the IPR rate, total number of coil procedures in ruptured cases, vascular territory (anterior cerebral artery, middle cerebral artery, posterior circulation) when available, study setting (tertiary/medical/oncological centers), and case accrual period.

Definitions and outcomes: The primary outcome was IPR rate per institution (IPR events divided by total coiling procedures). Annual provider volume was derived by dividing the number of reported cases by the study duration; high-volume centers were defined as those performing ≥20 ruptured aneurysm coiling procedures per year, and low-volume centers as those performing <20 [12,13].

Risk of bias and certainty: We assessed risk of bias and certainty of evidence with GRADE adapted for single-arm reviews, applying the core intent of each domain (Supplementary Material S3) [13]. Two reviewers (C.C. and K.C.) carried out GRADE judgments independently, and consensus was reached with F.T.

Synthesis and statistical analysis: We pooled IPR proportions separately for high- and low-volume centers using random effects models with maximum likelihood estimation of between-study variance. We conducted subgroup analyses by vascular territory and meta-regression of IPR rate versus provider volume (cases/year). We report 95% confidence intervals, consider *p* < 0.05 as indicating statistical significance, and interpret heterogeneity as follows: I^2^ < 40% unimportant, 30–60% moderate, 50–90% substantial, and 75–100% considerable. Analyses were conducted using R (R Development Core Team 2020, www.r-project.org) with the meta [14] and metafor packages. GRADE assessments were prepared using GRADEpro GDT.

Data, code, and materials availability: De-identified extracted datasets will be deposited in OSF with persistent accession links https://osf.io/bpkha (accessed on 8 July 2026). There are no restrictions on the materials or data availability.

## 3. Results

A total of 4581 studies were identified and screened (Figure 1), of which 618 studies were assessed for eligibility and 98 were included in the meta-analysis. We document the reasons for excluding 90 eligible studies in Supplementary Material S4, and the characteristics of the included studies, including individual citations, are detailed in Supplementary Material S6. Inter-reviewer agreement was assessed using Cohen’s kappa, yielding a value of 0.956 (98.86% agreement; Supplementary Material S5). Among the included studies, one was in Chinese [15], one in French [16], and one in Ukrainian [17]; the rest were in English.

**Figure 2:**
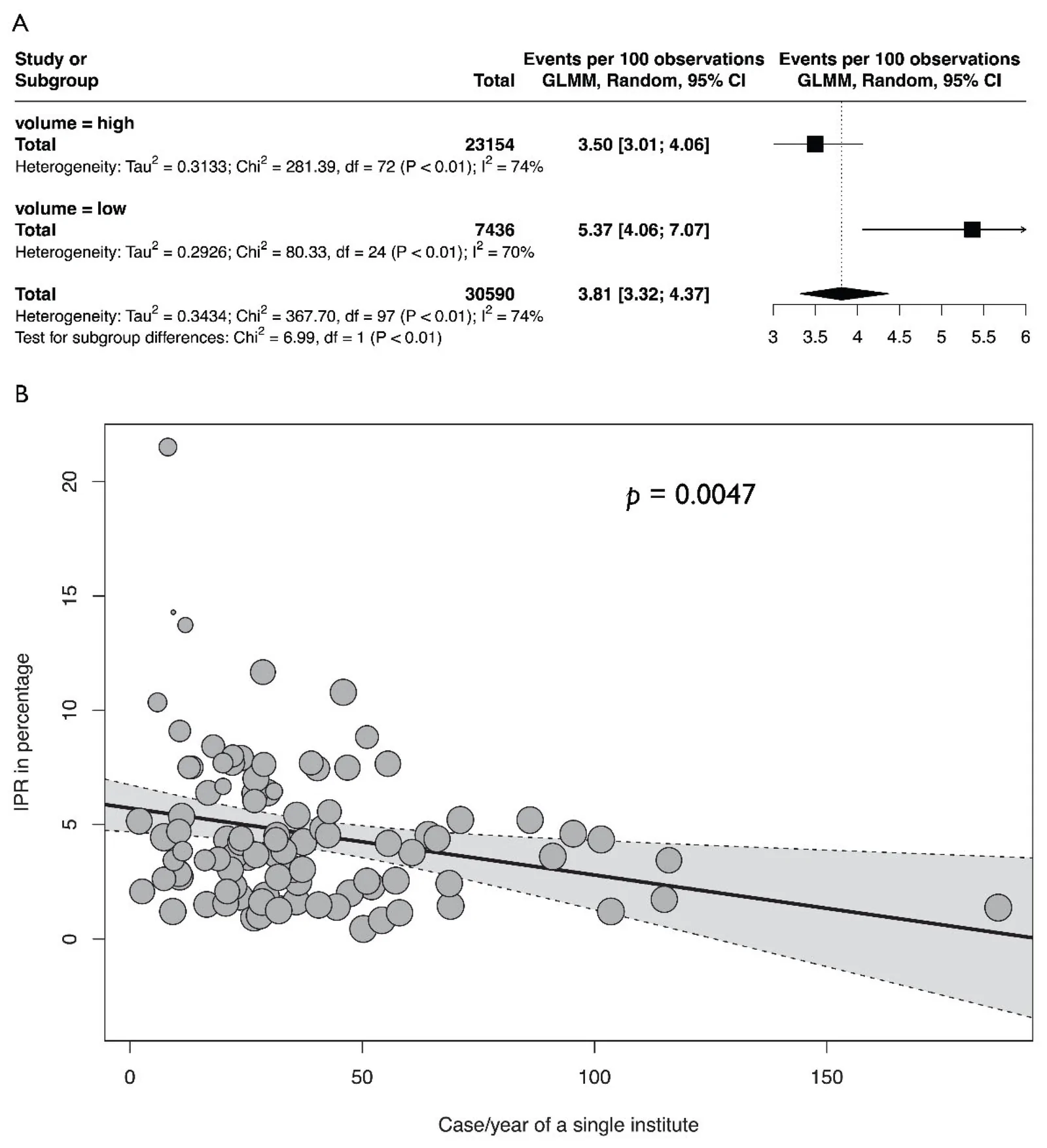
Intraprocedural rupture rates according to institutional provider volume. (**A**) Forest plot comparing intraprocedural rupture rates between high- and low-volume centers, using a generalized linear mixed model (GLMM). For each individual study, the effect estimate is represented by a red square. Arrows at the ends of a confidence interval line indicate that the CI extends beyond the plotted x-axis limits. (**B**) Meta-regression analysis demonstrating the association between institutional provider volume and intraprocedural rupture risk. The solid line represents the fitted regression line, indicating the predicted effect size as a function of the covariate. The dashed lines represent the 95% CI around the regression slope, illustrating the uncertainty of the estimated regression coefficient. The grey shaded area represents the 95% prediction interval for the true effect size at a given covariate value, reflecting the predicted dispersion of individual study effects. The size of each circle is proportional to the inverse variance (study weight), with larger circles denoting studies that contribute more statistical influence to the meta-regression model.

The summary of findings and the forest plot of IPR rates among high- and low-volume centers are presented in Table 1 and Figure 2A, respectively. The IPR rate in high-volume centers was 3.5% (95% CI: 3.01–4.06%; 23,154 participants; 73 studies; I^2^ = 74%); in contrast, that in low-volume centers was 5.37% (95% CI: 4.06–7.07%; 7436 participants; 25 studies; I^2^ = 70%). The difference between subgroups was statistically significant (*p* < 0.01). The full forest plot is provided in Supplementary Material S7. According to the GRADE criteria, the certainty of the evidence was classified as very low (GRADE definitions, downgrade criteria, and implications are provided in Supplementary Material S3).

Given that the number of cases per year for each center is a continuous variable, we conducted a meta-regression analysis (Figure 2B) to examine whether an increase in provider volume was associated with a reduction in the IPR rate. The analysis showed a significant association (*p* = 0.0047), with a regression coefficient of −0.65. While this indicates a statistical trend toward lower IPR rates with increasing volume, this coefficient should not be interpreted as a clinically applicable linear reduction in absolute risk. The relationship is observational and valid only within the observed volume distribution; extrapolation beyond this range or direct quantitative application of the coefficient to predict individual center outcomes is not appropriate.

### 3.1. Subgroup Analysis Based on Aneurysm Location

#### 3.1.1. Anterior Cerebral Artery

**Figure 3:**
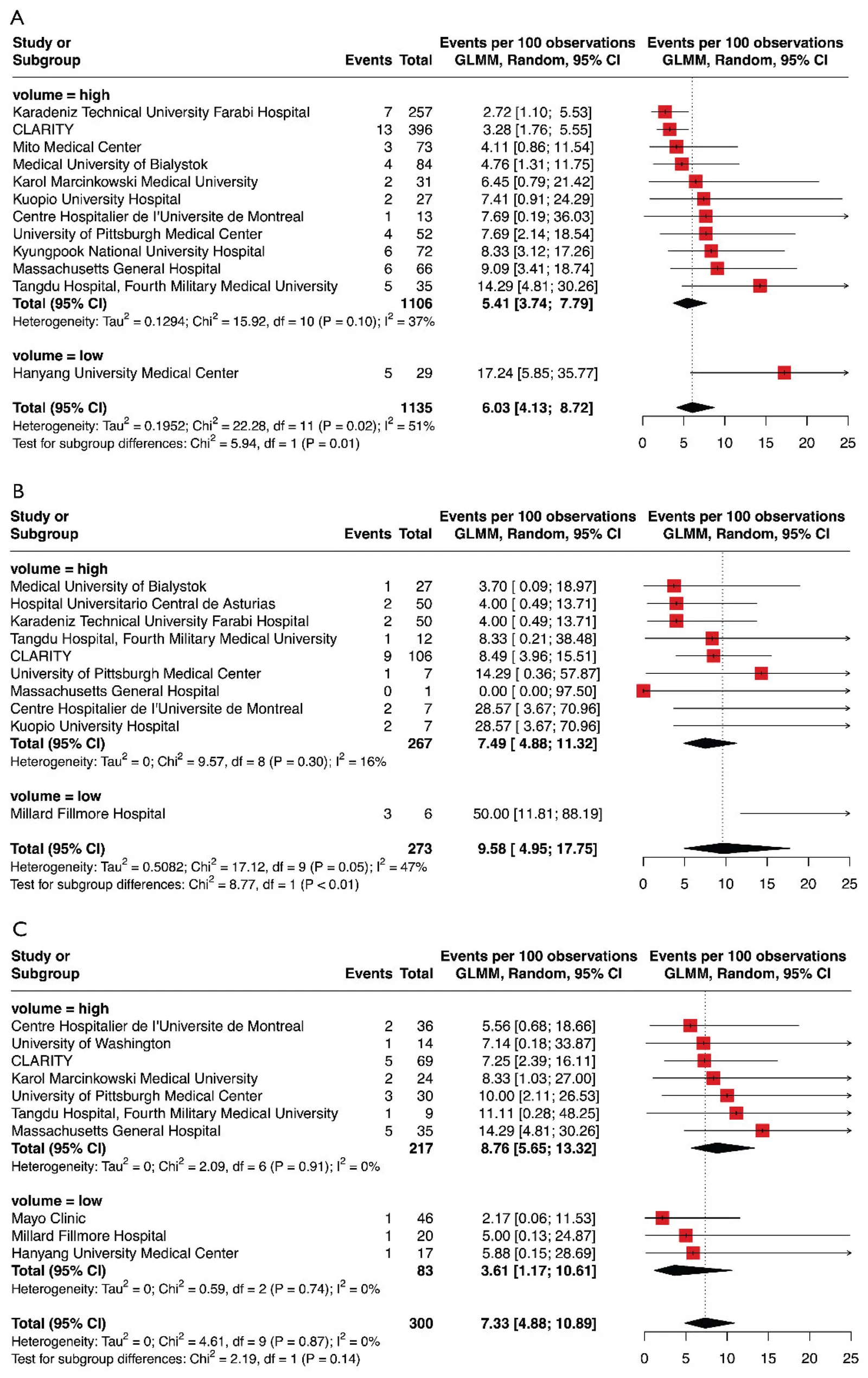
Intraprocedural rupture rates by aneurysm location and institutional volume. (**A**) Forest plot of intraprocedural rupture rates for anterior cerebral artery aneurysms in high-versus low-volume centers, using a generalized linear mixed model (GLMM). For each individual study, the effect estimate is represented by a red square. Arrows at the ends of a confidence interval line indicate that the CI extends beyond the plotted x-axis limits. (**B**) Forest plot of intraprocedural rupture rates for middle cerebral artery aneurysms in high-versus low-volume centers. (**C**) Forest plot of intraprocedural rupture rates for posterior circulation aneurysms in high- versus low-volume centers.

**Figure 4:**
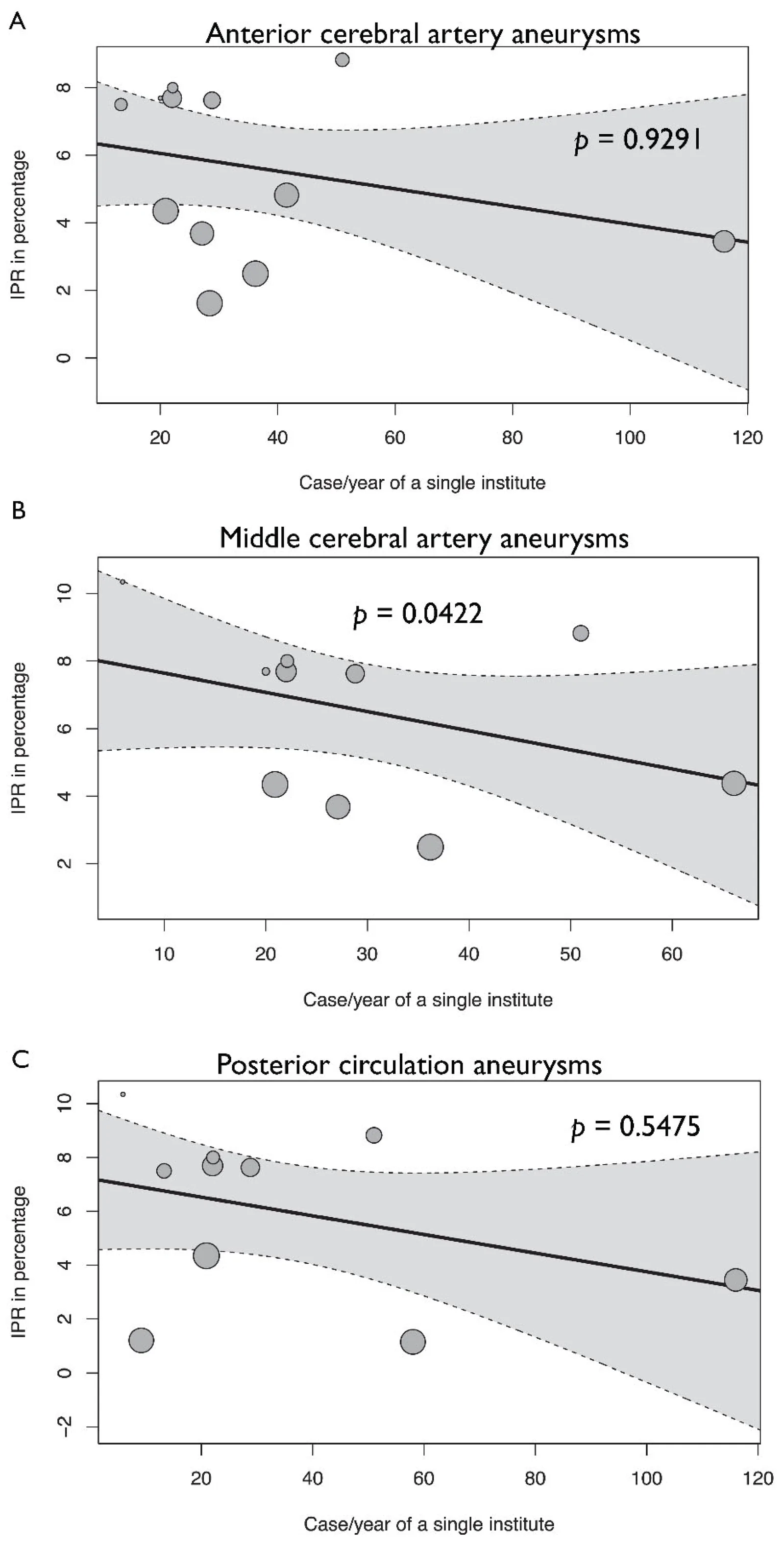
Meta-regression analyses stratified by aneurysm location. The solid line represents the fitted regression line, indicating the predicted effect size as a function of the covariate. The dashed lines represent the 95% CI around the regression slope, illustrating the uncertainty of the estimated regression coefficient. The grey shaded area represents the 95% prediction interval for the true effect size at a given covariate value, reflecting the predicted dispersion of individual study effects. The size of each circle is proportional to the inverse variance (study weight), with larger circles denoting studies that contribute more statistical influence to the meta-regression model. (**A**) Meta-regression analysis of the association between institutional provider volume and intraprocedural rupture risk for anterior cerebral artery aneurysms. (**B**) Meta-regression analysis of the association between institutional provider volume and intraprocedural rupture risk for middle cerebral artery aneurysms. (**C**) Meta-regression analysis of the association between institutional provider volume and intraprocedural rupture risk for posterior circulation aneurysms.

The IPR rate of anterior cerebral artery aneurysms among high- and low-volume centers is presented in Table 1 and Figure 3A, respectively. The IPR rate in high-volume centers was 5.41% (95% CI: 3.74–7.79%; 1106 participants; 11 studies; I^2^ = 37%); in contrast, the IPR rate in low-volume centers was only available from one study, at 17.24% (95% CI: 5.85–35.77%; 29 participants). The difference between subgroups was statistically significant (*p* = 0.01). However, the evidence was very low (Supplementary Material S3) and the meta-regression analysis (Figure 4A) yielded a *p*-value of 0.9291, indicating that a significant influence of provider volume on the IPR rate was not supported. Nevertheless, given that the low-volume group in this subgroup was represented by only a single study, this exploratory finding should be interpreted with caution and requires confirmation in future investigations.

#### 3.1.2. Middle Cerebral Artery

The IPR rate of middle cerebral artery aneurysms among high- and low-volume centers is presented in Table 1 and Figure 3B, respectively. The IPR rate in high-volume centers was 7.49% (95% CI: 4.88–11.32%; 267 participants; 9 studies; I^2^ = 16%); in contrast, the IPR rate in low-volume centers was only available from one study, at 50.0% (95% CI: 11.81–88.19%; 6 participants). The difference between subgroups was statistically significant (*p* < 0.01). Although the evidence was very low (Supplementary Material S3), the meta-regression analysis (Figure 4B) yielded a *p*-value of 0.0422, suggesting a statistically significant association between provider volume and IPR rate in this subgroup. However, given that the low-volume group consisted of only a single study with a very small sample size (*n* = 6), the regression coefficient should not be interpreted as a clinically applicable linear dose–response relationship. This finding should be viewed as an exploratory trend that requires cautious interpretation and validation in future studies with more balanced comparisons.

#### 3.1.3. Posterior Circulation

For posterior circulation aneurysms (Table 1 and Figure 3C), the IPR rate in high-volume centers was 8.76% (95% CI: 5.65–13.32%; 217 participants; 7 studies; I^2^ = 0%); in contrast, the IPR rate in low-volume centers was 3.61% (95% CI: 1.17–10.61%; 83 participants; 2 studies, I^2^ = 0%). The difference between subgroups was not statistically significant (*p* = 0.14); the evidence was very low (Supplementary Material S3), and the meta-regression analysis (Figure 4C) yielded a *p*-value of 0.5475, indicating that a significant influence of provider volume on the IPR rate was not supported.

## 4. Discussion

This systematic review and meta-analysis demonstrated that treatment at high-volume centers is associated with a significantly lower risk of IPR during endovascular coiling for ruptured intracranial aneurysms. Across nearly 100 studies including over 30,000 patients, high-volume centers (≥20 cases/year) had an IPR rate of 3.5%, compared with 5.37% in low-volume centers (<20 cases/year). This volume–outcome relationship persisted in subgroup analyses of anterior cerebral artery and middle cerebral artery aneurysms. Interestingly, the result was opposite for posterior circulation aneurysms. In addition, our meta-regression showed that increasing procedural volume was independently associated with a lower IPR rate, supporting a dose–response relationship between experience and safety.

Previous studies have demonstrated that higher hospital treatment volumes are associated with reduced mortality, lower rebleeding rates, and improved functional outcomes in patients with aneurysmal subarachnoid hemorrhage. Similarly, increased endovascular provider volume has been linked to fewer periprocedural complications in carotid artery stenting and mechanical thrombectomy. Our findings build on this evidence by suggesting an association between procedural volume and the IPR rate.

Inherent anatomical factors also contribute to variation in IPR rates across aneurysm locations. Previous studies have reported an IPR rate of approximately 4.2% for ACA aneurysms, with the anterior communicating artery identified as an independent risk factor—likely due to anatomical features such as a small dome size, the presence of basal outpouchings, and complex bifurcation geometry [15,16]. MCA aneurysms have shown an even higher IPR rate, reaching 4.8%, which may be attributed to their frequent wide-neck morphology and involvement of branch vessels, potentially complicating procedures such as balloon-assisted coiling or stent-assisted coiling [17,18,19]. For posterior circulation aneurysms, one study reported an IPR rate of 5.8%. This finding may partly reflect the anatomical and hemodynamic characteristics of posterior circulation aneurysms. However, this explanation remains speculative and should be interpreted cautiously given the limited available data [20,21].

A center’s procedural volume may reflect multiple dimensions of institutional capability, including operator skill, availability of adjunctive devices, and the maturity of perioperative protocols. These factors may contribute to the observed association between institutional volume and IPR rates. However, institutional volume should be considered a surrogate marker rather than an independent determinant of procedural safety.

Structured training, simulation-based skill development, and adherence to standardized coiling protocols may help improve procedural safety, particularly in lower-volume settings. Incorporating the IPR rate as a quality metric within endovascular registries may further facilitate benchmarking, guide targeted improvement efforts, and promote safer practice across institutions.

This study has several limitations. First, the included studies were all observational and mostly retrospective, introducing inherent risks of selection bias and confounding. The definition and reporting of IPR were not standardized, potentially contributing to heterogeneity. Second, procedural volume was derived from published data and may not reflect individual operator experience or temporal changes in practice. Third, patient- and aneurysm-level covariates, such as aneurysm size, neck configuration, and timing after rupture, were inconsistently reported. Moreover, institutional volume likely reflects multiple factors beyond procedural volume alone, including operator experience and institutional resources. Therefore, residual confounding cannot be excluded, and causality cannot be inferred from this observational single-arm meta-analysis. Finally, the certainty of evidence according to GRADE was rated as very low, highlighting the need for prospective, high-quality multicenter studies to validate these findings.

The subgroup analyses should also be interpreted with caution because the ACA and MCA comparisons each included only a single low-volume study, while the posterior circulation subgroup comprised a relatively small number of patients. Because this meta-analysis pooled event rates separately from single-arm studies, direct adjusted comparisons between high- and low-volume institutions were not possible.

Future research should incorporate patient-level data and standardized definitions of IPR to enable risk-adjusted analyses. Prospective registries could help in evaluating the relative contributions of institutional, operator, and case-mix factors to procedural safety. Additionally, research on learning curves, team-based training, and the impacts of mentorship programs may contribute to establishing minimum case thresholds for safe practice in endovascular coiling.

## 5. Conclusions

In summary, this meta-analysis demonstrated that treatment at high-volume centers (≥20 cases per year) may be associated with a lower risk of intraprocedural rupture during endovascular coiling of ruptured intracranial aneurysms. Increased procedural exposure and accumulated experience may contribute to improved procedural safety, particularly for aneurysms of the anterior and middle cerebral arteries. However, these findings should be interpreted with caution, given the observational nature of the included studies and the very low certainty of the available evidence. Further high-quality prospective studies are warranted to validate these findings and to better define the factors associated with improved procedural safety in this high-risk patient population.

## Figures and Tables

**Table 1:** Summary of findings. Impact of provider volume on intraprocedural rupture risk in endovascular treatment of ruptured intracranial aneurysms: A systematic review and meta-analysis. Patient or population: Ruptured intracranial aneurysms that underwent endovascular treatment. Setting: Tertiary neurovascular institutions that perform embolization of intracranial aneurysms. Definition of the data analyzed: intraprocedural rupture—new hemorrhage event within endovascular procedure.

Intraprocedural Rupture	Low Volume	High Volume	*p*-Value of Meta-Regression	No. of Participants (Studies)	Certainty of Evidence (GRADE)
All types of aneurysm per institute	Anticipated absolute rate (95% CI)		0.0047	30,590 (98 studies)	**⨁◯◯◯** **Very low**
	5.37 (4.06–7.07) per 100	**3.5** **(3.01–4.06)** **per 100**			
Anterior cerebral artery aneurysms	17.24 (5.85–35.77) per 100	**5.41** **(3.74–7.79)** **per 100**	0.9291	1135 (12 studies)	**⨁◯◯◯** **Very low**
Middle cerebral artery aneurysms	50 (11.81–88.19) per 100	**7.49** **(4.88–11.32)** **per 100**	0.0422	273 (10 studies)	**⨁◯◯◯** **Very low**
Posterior circulation aneurysms	3.61 (1.17–10.61) per 100	**8.76** **(5.65–13.32) per 100**	0.5475	300 (10 studies)	**⨁◯◯◯** **Very low**

CI: Confidence interval; GRADE: Grades of Recommendation, Assessment, Development, and Evaluation. GRADE Working Group grades of evidence: High certainty—we are very confident that the true effect lies close to that of the estimate of the effect. Moderate certainty—we are moderately confident in the effect estimate; the true effect is likely to be close to the estimate of the effect, but there is a possibility that it is substantially different. Low certainty—our confidence in the effect estimate is limited; the true effect may be substantially different from the estimate of the effect. Very low certainty—we have very little confidence in the effect estimate; the true effect is likely to be substantially different from the estimate of effect.

## Data Availability

The data presented in this study are available from the Open Science Framework repository (https://osf.io/bpkha (accessed on 8 July 2026)).

## Supplementary Materials

The following supporting information can be downloaded at: https://www.mdpi.com/article/10.3390/life16091393/s1.

## Abbreviations

The following abbreviations are used in this manuscript:

IPR	Intraprocedural rupture
ACA	Anterior cerebral artery
MCA	Middle cerebral artery
