# Supplementary material for: Impact of Provider Volume on Intraprocedural Rupture Risk During Endovascular Treatment of Ruptured Intracranial Aneurysms: A Systematic Review and Meta-Analysis
Source: Life (Basel). 2026 Aug 24;16(9):1393. doi: 10.3390/life16091393 (PMC13608014; doi:10.3390/life16091393)

SUPPLEMENTARY MATERIAL

Impact of provider volume on intraprocedural rupture risk in endovascular treatment of ruptured intracranial aneurysms: A systematic review and meta-analysis

Contents

Supplementary Material S1: Search Strategy for studies included in this review……….…..….……..…..……2

Supplementary Material S2: Data extraction form…………….……………………….…....….………………5

Supplementary Material S3: GRADE definitions and judgment process…………………….….……...…..….6

Supplementary Material S4: Reasons for excluding 90 eligible studies.…….….…….………..………...……10

Supplementary Material S5: Study selection and inter-assessor agreement…………………..…….………….17

Supplementary Material S6: Characteristics of included studies…...…………………………………………19

Supplementary Material S7: Full forest plot of IPR among high- and low-volume centers…..………………23

Supplementary Material S1. Search strategy for studies included in this review

The following search strategy, using a combination of MeSH terms and free text terms, was used for MEDLINE, Embase, Web of Sciences, and Google Scholar searches.

MEDLINE

Safari Version 16.6 (17615.3.12.11.3, 17615)

	10-September-2023	Results
1	Intracranial Aneurvsm/	31889
2	Aneurysm, Ruptured/	9866
3	Subarachnoid Hemorrhage/	23840
4	2 or 3	30752
5	1 and 4	11115
6	Embolization, Therapeutic/	36602
7	Endovascular Procedures/	27556
8	(intraoperati* rupture or intraoperati* he?morrhage or intraprocedur* rupture or intraprocedur* he?morrhage).ab,ti	1353
9	6 or 7 or 8	62391
10	5 and 9	2465
11	limit 10 to (humans and "all adult (19 plus years)")	1793

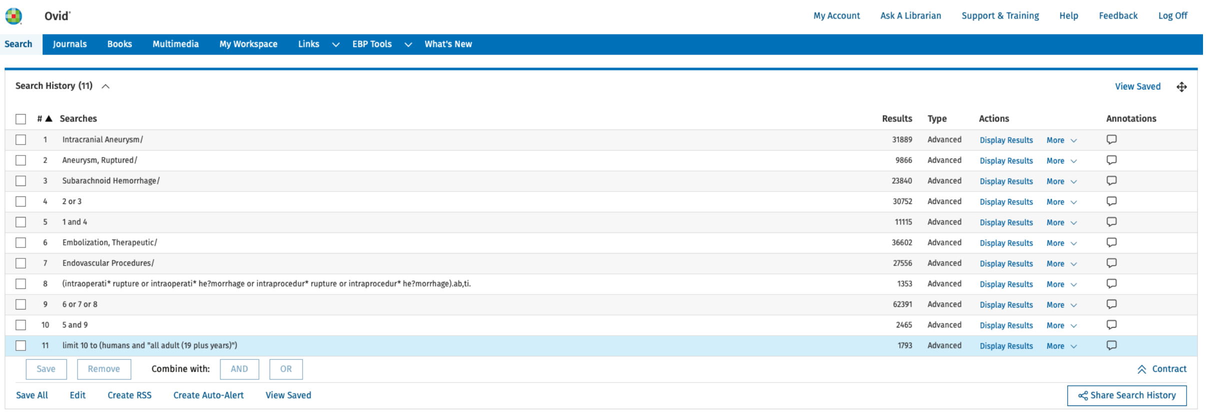

Embase

Safari Version 16.6 (17615.3.12.11.3, 17615)

	10-September-2023	Results
1	‘brain artery aneurysm rupture’	4003
2	‘intracranial aneurysm’	25458
3	‘subarachnoid hemorrhage’	59160
4	‘artificial embolization’	67172
5	‘coil embolization’	18677
6	‘endovascular aneurysm repair’	22522
7	‘endovascular surgery’	54596
8	‘intraoperative bleeding’	6090
9	‘intraoperative hemorrhage’	1186
10	‘peroperative complication’	62596
11	#1 OR #3	60753
12	#2 AND #11	8721
13	#4 OR #5 OR #6 OR #7 OR #8 OR #9 OR #10	211600
14	#12 AND #13	2470
15	#12 AND #13 AND ((article)/lim OR [article in press}/lim OR (preprint/lim) AND ([adult)/lim OR [aged)/lim) AND [humans]/im AND (clinical study|/lim AND (embase]/lim OR (preprint)/lim)	1379

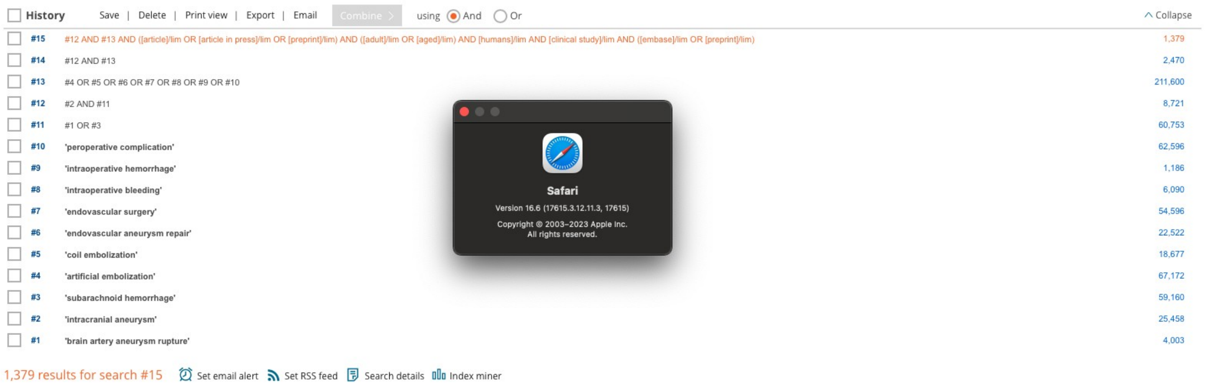

Web of Science
Safari Version 16.6 (17615.3.12.11.3, 17615)
11-September-2023

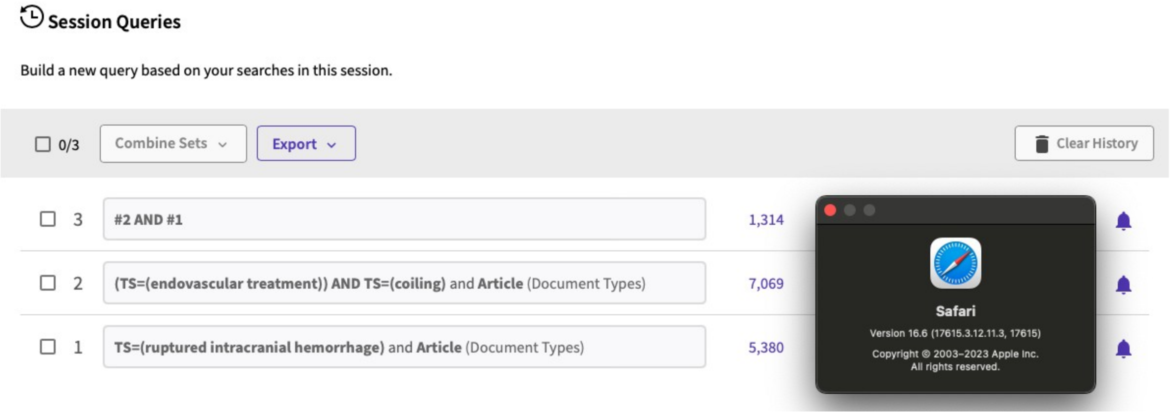

Google Scholar
Safari Version 16.6 (17615.3.12.11.3, 17615)
10-September-2023

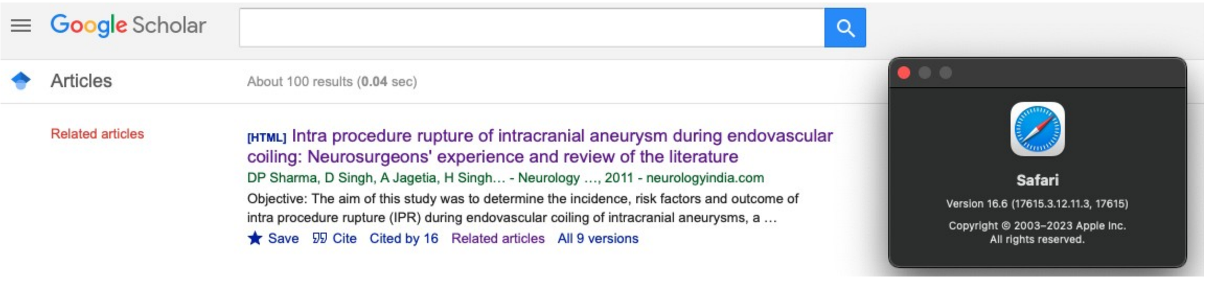

Supplementary Material S2. Data extraction form

Data Extraction Form 1.0

Study ID:____________________

Extracted by: ☐ Chen Kuo-Wei ☐ Chai Chung Liang ☐ Tsuang Fon-Yih

Citation:____
____
____
____

Population:

☐ Compatible with ruptured aneurysm underwent endovascular treatment
☐ Not-compatible

Intervention/Control:

Institute volume calculation
Total number of cases: n = ______
Duration of patient recruitment, in years:______
Number of institute within the publication:______
Patient per institute per year:______
☐ High-volume: ≥20 patient per institute per year
☐ Low-volume: <20 patient per institute per year

Outcome

event: n = ______ (type of endovascular treatment:________________________)
total population: n = ______

Subgroup/Moderator analysis

Final year of case recruitment:______

GET IT ON Google Play
Download on the App Store
怡仁綜合醫院 Yeezen General Hospital
326桃園市楊梅區楊新北路321巷30號
03-4855566
怡仁綜合醫院粉絲團
民眾意見反映電子信箱

Supplement Material S3. GRADE definitions and judgment process

Please refer to the respective references of GRADE in the main manuscript as all contents under this section are based on published guidelines and no alteration or modification of original definition or methodology has been made.

GRADE is an abbreviation of "Grades of Recommendation, Assessment, Development, and Evaluation.'' The complete formal description is in the GRADE handbook (see main manuscript for reference). There are five domains of GRADEs which is defined in the following:

Domains of GRADE	Definition
Risk of bias (study limitations)	There are several risk of bias in studies, especially in non-randomized studies, such as inadequate follow-up, failure to apply appropriate inclusion criteria, control confounding, and applying flawed measurement of exposure or outcome.
Publication bias	This domain focused on whether there is a selective publication of studies that may cause systematic under-estimation or an over-estimation of the underlying treatment effect. It is generally known that statistically significant studies are more likely to be published than insignificant studies.
Imprecision	Studies with small number of patients and low event rates are likely to be not accurate – imprecise. This can be evident when there is a wide confidence interval around the effect estimate.
Inconsistency	This means unexplained heterogeneity of results.
Indirectness	Revisit population, intervention, comparator, and outcomes (PICO) to judge whether the effect summary has a direct relationship with it.

Downgrade criteria to the 5 domains used in this study are summarized in the following:

Domains of GRADE	Criteria for downgrade
Risk of bias (study limitations)	In our study, we transfer the outcome of ROBINS-I into this domain.<br><br>a) Start as high, if outcome of ROBINS-I is low risk of bias<br>b) Start as moderate, if outcome of ROBINS-I is moderate risk of bias<br>c) Start as low, if outcome of ROBINS-I is serious risk of bias<br>d) Start as very low, if outcome of ROBINS-I is critical risk of bias<br><br>The complete formal description is in GRADE guideline number 18 (see main manuscript for reference)
Publication bias	Visual inspection of a funnel plot and statistical tests for asymmetry (if there are at least 5 studies included in the meta-analysis).<br>Rating down quality of evidence for publication bias by a maximum of one level.
Imprecision	Our study (systematic review) used Optimum Information Size (OIS) as the criteria for downgrading (GRADE has separate criteria for downgrading for guideline panels)

	For continuous outcomes<br>1)<br>Accordingly, downgrade the quality rating by one if serious or two levels if very serious.
Inconsistency	We use criteria below in the downgrade of this domain<br>1. Wide variance of point estimates across studies (note: direction of effect is not a criterion for inconsistency)<br>2. Minimal or no overlap of confidence intervals (CI), which suggests variation is more than what one would expect by chance alone<br>3. Statistical criteria, I^2:<br>● < 40% may be low<br>● 30-60% may be moderate<br>● 50-90% may be substantial<br>● 75-100% may be considerable<br>Accordingly, downgrade the quality rating by one if serious or two levels if very serious.
Indirectness	We use criteria below in the downgrade of this domain<br>1. Differences in population<br>● Indirectness in Populations (Downgraded by Two Levels)<br>● Non-human studies providing high quality evidence (Not Downgraded)<br>2. Differences in interventions<br>● Interventions delivered differently in different settings (Downgraded by One Level)<br>● Trials of related interventions (Downgraded by One or Two Levels)<br>● Indirectness in Interventions (Not Downgraded)<br>3. Differences in outcomes measures<br>● Time differences in outcomes (Downgraded by One Level)<br>● Surrogate outcomes (Downgraded by One or Two Levels)<br>● Uncertainty in the relationship between surrogate and Surrogate outcomes (Downgraded by One or Two Levels)<br>4. Indirect comparisons<br>● Indirect comparison (Downgraded by One Level)<br>● Network meta-analysis (Downgraded by Two Levels)<br>Accordingly, downgrade the quality rating by one if serious or two levels if very serious.

An outcome of the assessment is termed as the certainty of evidence, which reflects the systematic reviewer's judgement on the accuracy of an estimate of the effect:

High certainty	We are very confident that the true effect lies close to that of the estimate of the effect
Moderate certainty	We are moderately confident in the effect estimate:<br>The true effect is likely to be close to the estimate of the effect, but there is a possibility that it is substantially different
Low certainty	Our confidence in the effect estimate is limited:<br>The true effect may be substantially different from the estimate of the effect
Very low certainty	We have very little confidence in the effect estimate:<br>The true effect is likely to be substantially different from the estimate of effect.

References:

Holger Schünemann, Brożek Jan, Gordon Guyatt, Andrew Oxman. GRADE handbook for grading quality of evidence and strength of recommendations. Updated October 2013. The GRADE Working Group, 2013. [Internet]. Available from: https://gdt.gradepro.org/app/handbook/handbook.html

Detailed Processes of GRADE on all IPRs, anterior cerebral artery, middle cerebral artery, and posterior circulation

**GRADE criteria**	**Rating** (highlight one)	**Footnotes** (explain reasons for down- or upgrading)	**Certainty of Evidence** (highlight one)
**Outcome:**			
**Study design**	start with low certainty ratings for non-randomized studies		⊕⊕⊕⊕ High<br>⊕⊕⊕◯ Moderate<br>⊕⊕◯◯ Low<br>⊕◯◯◯ Very Low
**Risk of Bias** (use ROBINS-I)	No<br>serious (-1)<br>very serious (-2)		
**Inconsistency**	No<br>serious (-1)<br>very serious (-2)		
**Indirectness**	No<br>serious (-1)<br>very serious (-2)	Uses case numbers within a publication as a surrogate data	
**Imprecision**	No<br>serious (-1)<br>very serious (-2)		
**Publication Bias**	Undetected<br>Strongly suspected (-1)		
**Other (upgrading factors, highlight all that apply)**	Large effect (+1 or +2) Dose response (+1 or +2)<br>No Plausible confounding (+1 or +2)	We have not identified these factors that warrant an upgrade.	

Supplement Material S4. Reasons for excluding 90 eligible studies

	Reasons	Title of Article	Source publication	First author
1	Only patients with A1 aneurysms presented, unable to determine high- or low-volume based on data presented	Angiographic Characteristics and Endovascular Treatment of Anterior Cerebral Artery A1 Segment Aneurysms	World Neurosurg. 2017 Jan:97:551-556	Ying-Ying Zhang
2	Only patients with ACoA aneurysms presented, unable to determine high- or low-volume	Angiographic outcome after endovascular therapy for anterior communicating artery aneurysms: correlation with vascular morphological features.	Jpn J Radiol. 2012 Oct;30(8):624-7	Akihiro Uemura
3	Only patients with MCA aneurysms presented, unable to determine high- or low-volume	A shift from open to endovascular repair in the treatment of ruptured middle cerebral artery aneurysms: a single institution experience	Neuroradiology. 2023 Sep;65(9):1353-1361	Jose Danilo Bengzon Diestro
4	Only patients with Cerecyte coil presented, unable to determine high- or low-volume	Cerecyte coil trial: Procedural safety and clinical outcomes in patients with ruptured and unruptured intracranial aneurysms	AJNR Am J Neuroradiol. 2012 Mar;33(3):474-80.	S Coley
5	Only patients with PICA aneurysms presented, unable to determine high- or low-volume	Coil Embolization Results of the Ruptured Proximal Posteriori Inferior Cerebellar Artery Aneurysm: A Single-Center 10 Years' Experience.	World Neurosurg. 2018 Sep:117:e645-e652	Jonghoon Kim
6	Only patients with basilar aneurysms presented, unable to determine high- or low-volume	Comparative assessment of woven endobridge embolization and standard coil occlusion for the treatment of ruptured basilar tip aneurysms	Neuroradiology. 2023 Apr;65(4):765-773.	Erkan Celik
7	Only patients with ACoA aneurysms presented, unable to determine high- or low-volume	Comparison between outcomes of endovascular and surgical treatments of ruptured anterior communicating artery aneurysms	Br J Neurosurg. 2021 Jun;35(3):313-318	Lauren Harris
8	Only patients with MCA aneurysms presented, unable to determine high- or low-volume	Effect Analysis of Microsurgical Clipping and Endovascular Embolization for the Treatment of Middle Cerebral Artery Aneurysms	World Neurosurg. 2019 May:125:e1074-e1081.	Li Zhang
9	Only patients with posterior circulation aneurysms presented	Endovascular coil embolization of ruptured and unruptured posterior circulation aneurysms: review of a 10-year experience.	Neurosurgery. 2007 Apr;60(4):626-36	Aditya S Pandey
10	Only patients with DACA aneurysms presented, unable to determine high- or low-volume	Endovascular Management of Ruptured Distal Anterior Cerebral Artery (DACA) Aneurysms: A Retrospective Review Study.	World Neurosurg. 2017 Nov:107:588-596	Shakir Husain
11	Only patients with PICA aneurysms presented, unable to determine high- or low-volume	Endovascular management of ruptured distal posterior inferior cerebellar artery aneurysms: A retrospective cohort study.	Medicine (Baltimore). 2018 Dec;97(49):e13300	Xianyi Chen
12	Only patients with DACA aneurysms presented, unable to determine high- or low-volume	Endovascular Treatment for Ruptured Aneurysms at Distal Cerebral Arteries.	World Neurosurg. 2019 Mar:123:e387-e392	Jiang Zhou
13	Only patients with MCA aneurysms presented	Endovascular treatment of 346 middle cerebral artery aneurysms: results of a 16-year single-center experience.	Neurosurgery. 2015 Jan;76(1):54-60	Yasha Kadkhodayan
14	Only patients with posterior circulation aneurysms	Endovascular treatment of acutely ruptured and unruptured aneurysms of the	J Neurosurg . 1997	J Raymond

	presented	basilar bifurcation	Feb;86(2):211-9	
15	A particular subset of the patients within the whole institute is published. In this case, only elderly ones.	Endovascular treatment of intracranial aneurysms in elderly patients: a systematic review and meta-analysis.	Stroke. 2013 Jul;44(7):1897-902.	Carmelo L Sturiale
16	Only patients with ACoA aneurysms presented, unable to determine high- or low-volume	Endovascular treatment of anterior communicating artery aneurysms: Results of clinical and radiological outcome in Newcastle	Br J Neurosurg. 2003 Jun;17(3):278-86	T Elias
17	Only patients with ACoA aneurysms presented, unable to determine high- or low-volume	Endovascular treatment of anterior communicating artery aneurysms using Guglielmi detachable coils	Neuroradiology . 1996 Nov;38(8):800-5	J Moret
18	Only patients with posterior circulation aneurysms presented	Endovascular treatment of basilar tip aneurysms using electrolytically detachable coils.	J Neurosurg. 1996 Mar;84(3):393-9	C G McDougall
19	Only patients with MCA aneurysms presented, unable to determine high- or low-volume	Endovascular treatment of middle cerebral artery aneurysms as first option: a single center experience of 92 aneurysms.	AJNR Am J Neuroradiol. 2007 Sep;28(8):1567-72.	R S Quadros
20	Only patients with MCA aneurysms presented, unable to determine high- or low-volume	Endovascular Treatment of Middle Cerebral Artery Aneurysms for 120 Nonselected Patients: A Prospective Cohort Study	AJNR Am J Neuroradiol . 2014 Apr;35(4):715-20	B Gory
21	Only patients with MCA aneurysms presented, unable to determine high- or low-volume	Endovascular treatment of middle cerebral artery aneurysms – single-centre results	Pol J Radiol. 2020 Dec 20:85:e650-e656.	Paweł Brzegowy
22	Only patients with MCA aneurysms presented, unable to determine high- or low-volume	Endovascular treatment of middle cerebral artery aneurysms with detachable coils: angiographic and clinical outcomes in 115 consecutive patients	Neurosurgery. 2009 May;64(5):876-88;	Shuichi Suzuki
23	Only patients with posterior circulation aneurysms presented	Endovascular treatment of posterior circulation aneurysms	Br J Neurosurg. 2001 Feb;15(1):39-43	D Birchall
24	Only patients with posterior circulation aneurysms presented	Endovascular treatment of posterior circulation aneurysms by electrothrombosis using electrically detachable coils	J Neurosurg. 1992 Oct;77(4):515-24.	G Guglielmi
25	Only patients with ACoA aneurysms presented, unable to determine high- or low-volume	[Endovascular treatment of ruptured aneurysms located at anterior communicating artery complex: a sixty-six cases report].	Zhonghua Wai Ke Za Zhi. 2016 May 1;54(5):352-7.	Xia Li
26	Only patients with MCA aneurysms presented, unable to determine high- or low-volume	Endovascular Treatment of Ruptured Middle Cerebral Artery Bifurcation Aneurysms. A Retrospective Observational Study of Short- and Long-Term Follow-Up.	Cardiovasc Intervent Radiol. 2021 Apr;44(4):587-595	Florian Hagen
27	A particular subset of the patients within the whole institute is published. In this case, only elderly ones.	Interdisciplinary treatment of ruptured cerebral aneurysms in elderly patients	J Neurosurg. 2010 Jun;112(6):1200-7	François Proust
28	Only patients with posterior circulation aneurysms presented	Endovascular treatment of ruptured posterior circulation cerebral aneurysms. Clinical and angiographic outcomes.	Stroke. 2000 Jan;31(1):100-10	T E Lempert
29	Only patients with MCA aneurysms presented, unable	Endovascular treatment with coils of 149 middle cerebral artery berry aneurysms	Radiology. 2005 Nov;237(2):611-9.	Akira Iijima

	to determine high- or low-volume			
30	Only patients with DACA aneurysms presented, unable to determine high- or low-volume	Management of distal anterior cerebral artery aneurysms: A single institution retrospective analysis (1997-2005)	Neurosurgery. 2007 Nov;61(5):909-16	Aditya Pandey
31	Only patients with posterior circulation aneurysms presented	Operative or endovascular treatment of ruptured intracranial vertebral artery aneurysms?	Neuroradiology. 2000 Sep;42(9):685-91.	C Groden
32	Only patients with MCA aneurysms presented, unable to determine high- or low-volume	Outcome and Prognostic Factors of Ruptured Middle Cerebral Artery Aneurysms Treated via Endovascular Approach: A Single-Center 11-Year Experience.	World Neurosurg. 2020 Jan:133:e187-e196.	Yu Zhou
33	Only patients with ACoA aneurysms presented, unable to determine high- or low-volume	Outcomes analysis of ruptured distal anterior cerebral artery aneurysms treated by endosaccular embolization and surgical clipping.	Interv Neuroradiol . 2011 Mar;17(1):49-57.	S Suzuki
34	Only patients with ACoA aneurysms presented, unable to determine high- or low-volume	Predicting intraprocedural rupture and thrombus formation during coiling of ruptured anterior communicating artery aneurysms.	J Neurointerv Surg. 2017 Apr;9(4):370-375	Lianghao Fan
35	Only patients with ACoA aneurysms presented, unable to determine high- or low-volume	Risk factors for intraprocedural rupture during emergency endovascular treatment of ruptured anterior communicating artery aneurysms	Interv Neuroradiol. 2022 Aug;28(4):426-432	Wei Shang
36	Only patients with ACoA aneurysms presented, unable to determine high- or low-volume	Safety and efficacy of different therapeutic strategies in the endovascular treatment of anterior cerebral artery aneurysms with different features: A single centre experience.	Clin Neurol Neurosurg . 2020 Jun:193:105786	Bin Yuan
37	Only patients with ACoA aneurysms presented, unable to determine high- or low-volume	Stand-alone coil embolization of anterior communicating artery aneurysms: Efficacy and technical issues.	Neuroradiol J. 2016 Oct;29(5):361-7	Damian Kocur
38	Only patients with posterior circulation aneurysms presented	Superior cerebellar artery aneurysms: incidence, clinical presentation and midterm outcome of endovascular treatment.	Neuroradiology. 2007 Sep;49(9):747-51	Jo P P Peluso
39	Only patients with posterior circulation aneurysms presented	Treatment of basilar artery bifurcation aneurysms by using Guglielmi detachable coils: a 6-year experience.	J Neurosurg. 1999 May;90(5):843-52	G Bavinzski
40	Only patients with middle cerebral artery presented, unable to determine high- or low- volume based on data presented	Treatment of ruptured middle cerebral artery aneurysms by endovascular approach: a single-center experience.	Int J Neurosci. 2017 May;127(5):433-438.	Xintong Zhao
41	A particular subset of the patients within the whole institute is published. In this case, only balloon-assisted ones.	Balloon-assisted coiling of cerebral aneurysms with the dual-lumen Scepter XC balloon catheter	Interv Neuroradiol. 2019 Aug;25(4):414-418.	Adam N Wallace
42	A particular subset of the patients within the whole institute is published. In this case, only wide-necked aneurysms.	Comparison of Stent-Assisted Coiling and Balloon-Assisted Coiling in the Treatment of Ruptured Wide-Necked Intracranial Aneurysms in the Acute Period.	World Neurosurg. 2016 Dec:96:316-321	Kefu Cai
43	A particular subset of the patients within the whole	Clinical Study on Complications of Intracranial Ruptured Aneurysm	Med Sci Monit. 2018 Nov	Yinlong Liu

	institute is published. In this case, only stent-assisted ones.	Embolization by Stent-Assisted Coil.	12:24:8115-8124	
44	Only patients with poor-grade presented	One-stage embolization in patients with acutely ruptured poor-grade aneurysm.	Surg Neurol. 2005 Feb;63(2):149-54	Yong Sam Shin
45	Only patients with poor-grade presented	Stent-assisted coiling versus coiling alone of poor-grade ruptured intracranial aneurysms: a multicenter study	J Neurointerv Surg. 2017 Feb;9(2):165-168	Bing Zhao
46	A particular subset of the patients within the whole institute is published. In this case, only elderly patients.	Endovascular treatment of ruptured intracranial aneurysms in elderly patients: clinical features and treatment outcome.	Neurosurg Rev. 2019 Sep;42(3):745-751.	Xiangke Ma
47	A particular subset of the patients within the whole institute is published. In this case, only wide-necked aneurysms.	Stent-assisted coiling of ruptured wide-necked intracranial aneurysms: A single-center experience of 218 consecutive patients	Neurol India . 2016 Mar-Apr:64 Suppl:S70-7	Peng Liu
48	A particular subset of the patients within the whole institute is published. In this case, only stent-assisted ones.	Safety and efficacy of intravenous tirofiban as antiplatelet premedication for stent-assisted coiling in acutely ruptured intracranial aneurysms	AJNR Am J Neuroradiol. 2016 Mar;37(3):508-14	S Kim
49	A particular subset of the patients within the whole institute is published. In this case, only elderly patients.	Endovascular management for intracranial ruptured aneurysms in elderly patients: Outcome and technical aspects	Neuroradiology. 2005 Jun;47(6):446-57.	F Mont'alverne
50	A particular subset of the patients within the whole institute is published. In this case, by a particular device.	GDC 18 treatment of ruptured and non-ruptured aneurysms	J Neuroradiol. 2007 May;34(2):89-94	S Gallas
51	Only patients with DACA aneurysms presented, unable to determine high- or low-volume	Endovascular treatment of ruptured distal anterior cerebral artery aneurysm.	Neurol India. 2010 Mar-Apr;58(2):259-63	Qinghai Huang
52	A particular subset of the patients within the whole institute is published. In this case, only wide-necked aneurysms.	Stent-assisted Coil Placement for the Treatment of 211 Acutely Ruptured Wide-necked Intracranial Aneurysms: A Single-Center 11-Year Experience.	Radiology. 2015 Aug;276(2):545-52.	Pengfei Yang
53	A particular subset of the patients within the whole institute is published. In this case, by a particular device.	Endovascular treatment of intracranial aneurysms with Barricade coils: Feasibility, procedural safety, and immediate postoperative anatomical results.	J Neuroradiol. 2016 Oct;43(5):353-7	Mohamed Zidan
54	A particular subset of the patients within the whole institute is published. In this case, only wide-necked aneurysms.	Modified balloon-assisted coiling instead of acute stenting in the treatment of ruptured wide necked intracranial aneurysms	Interv Neuroradiol. 2022 Jun;28(3):338-346	Cetin Murat Altay
55	A particular subset of the patients within the whole institute is published. In this case, only wide-necked aneurysms.	Safety and feasibility of tirofiban used for intracranial ruptured cystic aneurysms during perioperative interventional treatment	Chin J cerebrovasc Dis, Aug 18, 2022 Vol. 19, No. 8	Chao Lyu
56	A particular subset of the patients within the whole institute is published. In this case, only stent-assisted ones.	Stent-assisted coiling versus coiling alone of ruptured anterior communicating artery aneurysms: A single-center experience.	Clin Neurol Neurosurg. 2016 May:144:96-100	Lianghao Fan
57	A particular subset of the patients within the whole institute is published. In this	Endovascular treatment of intracranial wide-necked aneurysms with GDCs combined with balloon or stent	Neuroradiol J. 2009 Mar 23;22(1):86-91	N Xu

	case, only wide-necked aneurysms.			
58	A particular subset of the patients within the whole institute is published. In this case, only wide-necked aneurysms.	Complications and adverse events associated with Neuroform stent-assisted coiling of wide-neck intracranial aneurysms	Neurol Res . 2011 Oct;33(8):841-52	Xu Gao
59	A particular subset of the patients within the whole institute is published. In this case, only elderly patients.	Endovascular treatment of ruptured intracranial aneurysms using platinum coils in patients over 70 years of age	Interv Neuroradiol. 2000 Nov 30;6 Suppl 1(Suppl 1):85-7.	M Sawada
60	A particular subset of the patients within the whole institute is published. In this case, by a particular device.	Stent-assisted coil embolization of intracranial aneurysms using the Solitaire™ AB Neurovascular Remodeling Device: Initial and midterm follow-up results	Neuroradiology. 2013 May;55(5):629-38.	Christin Clajus
61	A particular subset of the patients within the whole institute is published. In this case, only wide-necked aneurysms.	Dual microcatheter coil embolization of acutely ruptured wide-necked intracranial aneurysms.	Interv Neuroradiol. 2017 Oct;23(5):477-484	Pyeong-Ho Yoon
62	A particular subset of the patients within the whole institute is published. In this case, only wide-necked aneurysms.	Treatment of ruptured intracranial aneurysms: comparison of stenting and balloon remodeling.	Neurosurgery. 2013 Jun;72(6):953-9.	Rohan Chitale
63	A particular subset of the patients within the whole institute is published. In this case, only elderly patients.	The impact of microsurgical clipping and endovascular coiling on the outcome of cerebral aneurysms in patients over 60 years of age.	J Clin Neurosci. 2012 Aug;19(8):1115-8	Qing-Rong Zhang
64	Only patients with DACA aneurysms presented, unable to determine high- or low-volume	Feasibility and efficacy of endovascular therapy for ruptured distal anterior cerebral artery aneurysms.	Neurol Med Chir (Tokyo) . 2013;53(5):304-9	Hidenori Oishi
65	Only patients with ACoA aneurysms presented, unable to determine high- or low-volume	“Coil mainly” policy in management of intracranial ACoA aneurysms: single-centre experience with the systematic review of literature and meta-analysis	Neurosurg Rev. 2018 Jul;41(3):825-839	Anna Steklacova
66	Only patients with poor-grade presented	Outcome in Poor Grade Subarachnoid Hemorrhage Patients Treated with Acute Endovascular Coiling of Aneurysms and Aggressive Intensive Care	Neurocrit Care . 2011 Jun;14(3):341-7.	Christopher J Taylor
67	Only patients with poor-grade presented	Is a high initial World Federation of Neurosurgery (WFNS) grade really associated with a poor clinical outcome in elderly patients with ruptured intracranial aneurysms treated with coiling?	J Neurointerv Surg. 2014 May;6(4):286-90.	Christina Iosif
68	A particular subset of the patients within the whole institute is published. In this case, by a particular device.	Semi-jailing technique using a Neuroform3 stent for coiling of wide-necked intracranial aneurysms	J Korean Neurosurg Soc. 2017 Mar;60(2):146-154.	Jun Kyeung Ko
69	A particular subset of the patients within the whole institute is published. In this case, only elderly patients.	Endovascular Treatment of Ruptured Intracranial Aneurysms in Elderly People	AJNR Am J Neuroradiol . 2004 Apr;25(4):592-5.	Boris Lubicz
70	A particular subset of the patients within the whole	Balloon-Assisted Coiling of Intracranial Aneurysms: Technical Details and	Neurol India. 2022 Mar-	S Vignesh

	institute is published. In this case, by a particular device.	Evaluation of Local Complications	Apr;70(2):643-651	
71	A particular subset of the patients within the whole institute is published. In this case, by a particular device.	The safety and efficacy of the LVIS stent for the treatment of ruptured intracranial aneurysms within 24hours: A multicenter retrospective study.	Clin Neurol Neurosurg. 2020 Oct:197:106117.	Xianggan Wang
72	A particular subset of the patients within the whole institute is published. In this case, by a particular device.	Balloon-assisted coiling of intracranial aneurysms using the Eclipse 2L double lumen balloon	Interv Neuroradiol. 2020 Jun;26(3):291-299	Raoul Pop
73	A particular subset of the patients within the whole institute is published. In this case, only elderly patients.	Endovascular embolization of ruptured cerebral aneurysms in patients older than 70 years.	J Clin Neurosci. 2007 Feb;14(2):127-32	Chao-Bao Luo
74	A particular subset of the patients within the whole institute is published. In this case, only wide-necked aneurysms.	Wide-necked intracranial aneurysms: treatment with stent-assisted coil embolization during acute (<72 hours) subarachnoid hemorrhage - experience in 61 consecutive patients.	Radiology. 2009 Oct;253(1):199-208	Olli I Tähtinen
75	A particular subset of the patients within the whole institute is published. In this case, only elderly patients.	Stent-assisted coiling of wide-necked aneurysms in the setting of acute subarachnoid hemorrhage: experience in 65 patients.	Neurosurgery. 2012 Jun;70(6):1415-29; discussion 1429	Peter S Amenta
76	A particular subset of the patients within the whole institute is published. In this case, only elderly patients.	Endovascular treatment of ruptured intracranial aneurysms in patients 70 years of age and older	Surg Neurol Int. 2014 Jul 5:5:104	Daisuke Watanabe
77	A particular subset of the patients within the whole institute is published. In this case, by a particular device.	Utility of low-profile visualized intraluminal support (LVIS (TM)) stent for treatment of acutely ruptured bifurcation aneurysms: A single-center study	Front Neurol . 2023 Mar 22:14:1050369	Changya Liu
78	Only patients with poor-grade presented	Outcome after endovascular treatment of hunt and hess grade IV or V aneurysms - Comparison of anterior versus posterior circulation	Stroke. 1999 Dec;30(12):2617-22	C Kremer
79	Only patients with MCA aneurysms presented, unable to determine high- or low-volume	Clipping Versus Coiling for Ruptured MCA Aneurysms Associated with Intracerebral Hematoma Requiring Surgical Evacuation	Neurocrit Care. 2023 Aug;39(1):162-171.	Thomas Metayer
80	A particular subset of the patients within the whole institute is published. In this case, only stent-assisted ones.	Stent-assisted treatment of ruptured intracranial aneurysms in the acute phase: A single center experience	eNeurologicalSci . 2018 Jan 28:10:31-36	Michael J Ho
81	A particular subset of the patients within the whole institute is published. In this case, only wide-necked aneurysms.	Results of Temporary Stent-assisted Coil Embolization (CATS) for the Treatment of Wide-neck Aneurysms: A 10-year Single Center Experience	Clin Neuroradiol. 2023 Mar;33(1):219-226	F Gottmann
82	Only patients with poor-grade presented	Contribution of endovascular therapy to the management of poor-grade aneurysmal subarachnoid hemorrhage	J Neurosurg. 2006 Nov;105(5):664-70	Shuichi Suzuki
83	A particular subset of the patients within the whole institute is published. In this case, only wide-necked aneurysms.	Safety of tirofiban and dual antiplatelet therapy in treating intracranial aneurysms	Stroke Vasc Neurol. 2019 Feb 3;4(1):36-42.	Edgar A Samaniego
84	A particular subset of the patients within the whole	Anatomic results and complications of stent-assisted coil embolization of	Interv Neuroradiol. 2008 Sep	S R Kim

	institute is published. In this case, only stent-assisted ones.	intracranial aneurysms	30;14(3):267-84	
85	A particular subset of the patients within the whole institute is published. In this case, by a particular device.	Neuroform Atlas stent-assisted coiling of ruptured intracranial aneurysms: A multicenter study	J Neuroradiol. 2021 Nov;48(6):479-485.	Riccardo Russo
86	A particular subset of the patients within the whole institute is published. In this case, only wide-necked aneurysms.	Safety and efficacy of a new prophylactic tirofiban protocol without oral intraoperative antiplatelet therapy for endovascular treatment of ruptured intracranial aneurysms.	J Neurointerv Surg. 2016 Nov;8(11):1148-1153	Xiao-Dong Liang
87	Only patients with middle cerebral artery presented, unable to determine high- or low- volume based on data presented	Angiographic and Clinical Results after Endovascular Treatment for Middle Cerebral Artery Berry Aneurysms	Neuroradiol J. 2007 Feb 28;20(1):89-101	N Hirota
88	Only patients with middle cerebral artery presented, unable to determine high- or low- volume based on data presented	Endovascular treatment of 174 middle cerebral artery aneurysms: clinical outcome and radiologic results at long-term follow-up	Radiology . 2009 Oct;253(1):191-8	Jean-François Vendrell
89	Only patients with anterior cerebral artery presented, unable to determine high- or low- volume based on data presented	Endovascular treatment of 429 anterior communicating artery aneurysms using bare-platinum coils: clinical and radiologic outcomes at the long- term follow-up	J Korean Neurosurg Soc. 2015 Mar;57(3):159-66.	Jong Young Lee
90	We cannot determine how many institutes involved in the study. Hence, unable to determine high- or low-volume	Factors related to adverse events during endovascular coil embolization for ruptured cerebral aneurysms	J Neurointerv Surg. 2020 Jun;12(6):605-609.	Hirotoshi Imamura

Supplement Material S5. Study selection and inter-assessor agreement

1st Reviewer: Kuo-Wei Chen
2nd Reviewer: Chung Liang Chai
3rd Reviewer: Fon-Yih Tsuang

Each reviewer works independently, and their differences in this inclusion or exclusion of studies were compared. Kuo-Wei Chen reported the process with the inter-assessor reliability with Cohen's kappa index to Chung Liang Chai. All disagreements and inaccuracies were resolved and monitored by Fon-Yih Tsuang. The Cohen's kappa was (% agreement) indicated moderate agreement. As reported in the following tables, no further disagreement was found after discussion between the reviewers. Please see the table below for the discussion. Please see the table below for the discussion.

Cohen's kappa is 0.956 (98.86% agreement)

n = 613		Chen's decision	
		Inclusion	Not to include
Chai's decision	Inclusion	91	3
	Not to include	4	515

Only **Chen** wanted to include the studies in the meta-analysis

Final Decision	Study Name	Discussion Process	Comments by Tsuang
Exclude	Factors related to adverse events during endovascular coil embolization for ruptured cerebral aneurysms<br><br>J Neurointerv Surg. 2020 Jun;12(6):605-609.	Chen: The IPR is compatible with our protocol<br><br>Chai: The paper does not indicate how many institute participated in the JR-NET3	We cannot determine whether JR-NET 3 is high or low volume
Exclude	Safety of tirofiban and dual antiplatelet therapy in treating intracranial aneurysms<br><br>Stroke Vasc Neurol. 2019 Feb 3;4(1):36-42.	Chen: The IPR is compatible with our protocol<br><br>Chai: They only studied stent-assisted coiling (SAC) or flow diversion treatments	Agree with Chai
Exclude	Five to ten years follow-up after coiling of 241 patients with acutely ruptured aneurysms: A single centre experience<br><br>Interv Neuroradiol. 2012 Mar;18(1):5-13	Chen: All criteria is compatible<br><br>Chai: Same data from another study from the same institute: Careggi University Hospital (Interv Neuroradiol . 2013 Jun;19(2):195-202.)	Regard as cohort duplicate from the same institute

Only **Chai** want to include the studies in the meta-analysis

Final Decision	Study Name	Discussion Process	Comments by Tsuang
Exclude	Ruptured intracranial aneurysms with small basal outpouching: incidence of basal rupture and results of surgical and endovascular treatments.<br><br>Neurosurgery. 2012 Nov;71(5):994-1001	Chai: A bit overlaps with another study but from the same institute<br><br>Chen: Should be regarded as a duplicate.	The study should be excluded, but the data is regarded from the same institute and merged with Kyungpook National University Hospital
Exclude	Safety and feasibility of tirofiban used for intracranial ruptured cystic aneurysms during perioperative interventional treatment<br><br>Chin J cerebrovasc Dis, Aug 18, 2022 Vol. 19, No. 8	Chai: The population is compatible<br><br>Chen: Only patients with wide-necked needing stents are considering tirofiban. It indirectly indicate a subset of patients instead of the whole institute.	Agree with Chen
Include	Aneurysmal subarachnoid hemorrhage in elderly patients: long-term outcome and prognostic factors in an interdisciplinary treatment approach<br><br>Neurol. 2013 Apr;260(4):1052-60.	Chai: The data is compatible with our protocol<br><br>Chen: The rupture rate is not normal	Should include because no violation of protocol.
Include	Procedure-related Complications during Endovascular Treatment of Intracranial Saccular Aneurysms<br><br>J Cerebrovasc Endovasc Neurosurg. 2017 September;19(3):162-170	Chai: The data is compatible with our protocol<br><br>Chen: The rupture rate is not normal	Should include because no violation of protocol.

Supplement Material S6. Characteristics of included studies.

Institute/ Collaborations	Country/ Region	Embolization methods	Total n. of cases	Institute involved	Case per institute per year	High volume (≥20)
Alfried Krupp Krankenhaus	Germany	simple coil	1034	1	101.4	yes
ARETA	France	simple coil or balloon assisted	753	16	31.4	yes
Azienda Ospedaliero-Universitaria Policlinico Umberto I	Italy	simple coil or balloon assisted	215	3	23.9	yes
Beth Israel Hyman-Newman Institute	United States of America	simple coil	48	1	20.9	yes
Beth Israel Hyman-Newman Institute	United States of America	simple coil	414	1	69	yes
CARAT	United States of America	simple coil	299	9	11.1	no
Careggi University Hospital	Italy	simple coil	510	1	45.9	yes
Centre Hospitalier de l'Universite de Montreal	Canada	simple coil	75	1	22.1	yes
Centre Hospitalier Universitaire Nice	France	simple coil	195	1	27.9	yes
Centre Hospitalier Universitaire Bretonneau	France	simple coil	683	5	25.2	yes
Changhai Hospital	China	simple coil	520	1	52	yes
Changhai Hospital	China	simple coil or stent assisted	414	1	103.5	yes
CHR de la Citadelle	Belgium	simple coil or balloon assisted	26	1	11.3	no
CHU de Bicêtre	France	simple coil or balloon assisted	320	1	26.7	yes
CLARITY	France	simple coil or stent assisted	782	20	20.9	yes
Clinical Center of Serbia	Serbia	simple coil	206	1	68.7	yes
Dr. Ram Manohar Lohia Institute of Medical Sciences	India	simple coil	90	1	42.9	yes
Enfant-Jésus Hospital	Canada	simple coil	532	1	29.6	yes
First Affiliated Hospital of China Medical University	China	simple coil or stent assisted	370	1	60.7	yes
First Affiliated Hospital of Dalian Medical University	China	simple coil or stent assisted	279	1	25.4	yes
First Hospital Hebei	China	simple coil or	244	1	54.2	yes

Medical University		stent assisted				
Fondation Ophtalmologique Adolphe de Rothschild	France	simple coil	132	1	42.6	yes
Frenchay Hospital	United Kingdom	simple coil	711	1	71.1	yes
G B Pant Hospital	India	simple coil	132	1	21.6	yes
GEL THE NEC	Five countries	simple coil or balloon assisted	214	27	1.98	no
Gold Coast Hospital	Australia	simple coil	60	1	20	yes
Guro Hospital	Korea	simple coil	124	1	20.6	yes
Hanyang University Medical Center	Korea	simple coil or stent assisted	80	1	13.3	no
Haukeland University Hospital	Norway	simple coil	83	1	26.8	yes
Hopital Erasme	Belgium	simple coil or stent assisted	457	1	32	yes
Hôpital Henri Mondor	France	simple coil or balloon assisted	91	1	23.9	yes
Hôpital Purpan, University of Toulouse	France	simple coil or balloon assisted	222	1	55.5	yes
Hospital Clínico San Carlos	Spain	simple coil	66	1	16.5	no
Hospital Universitario Central de Asturias	Spain	simple coil or balloon assisted	259	1	66.07	yes
Huashan Hospital	China	simple coil or stent assisted	637	1	91	yes
ISAT	Europe and North America	simple coil	1073	16	16.8	no
ISAT-2	United States, Canada, Spain	simple coil	48	4	2.6	no
JR-NET	Japan	simple coil or balloon assisted	5102	139.2	7.3	no
Karadeniz Technical University Farabi Hospital	Turkey	simple coil or balloon assisted	481	1	36.2	yes
Karol Marcinkowski Medical University	Poland	simple coil	116	1	116	yes
Klinikum Stuttgart	Germany	simple coil	452	1	50.2	yes
Kokura Memorial Hospital	Japan	simple coil	28	1	9.3	no
Konyang University Hospital	Korea	simple coil or stent assisted	185	1	31.9	yes
Kuopio University Hospital	Finland	simple coil	52	1	20	yes
Kurashiki Central Hospital	Japan	simple coil or balloon assisted	117	1	39	yes
Kyoto Prefectural Yosanoumi Hospital	Japan	simple coil	38	1	7.3	no
Kyungpook National University Hospital	Korea	simple coil or stent assisted	332	1	41.5	yes
Lariboisière Hospital	France	simple coil	158	1	51	yes

Ludwig-Maximilians-University	Germany	simple coil	93	1	8.14	no
Massachusetts General Hospital	United States of America	simple coil	156	1	22	yes
Mayo Clinic	United States of America	simple coil or balloon assisted	83	1	9.2	no
Medical University of Bialystok	Poland	simple coil or balloon assisted	190	1	27.1	yes
Medical University of Silesia	Poland	simple coil	77	1	10.7	no
Millard Fillmore Hospital	United States of America	simple coil	58	1	5.9	no
Mito Medical Center	Japan	simple coil	247	1	28.4	yes
Neurosurgical Centre Nijmegen	Netherlands	simple coil	285	1	44.5	yes
New York Presbyterian Hospital	United States of America	simple coil or balloon assisted	352	1	35.9	yes
Okayama University Medical School	Japan	simple coil	134	1	40.6	yes
Oregon Health Sciences University	United States of America	simple coil	31	1	31	no
Prince of Wales Hospital	Hong Kong	simple coil	80	1	12.7	no
Qilu Hospital of Shandong University	China	simple coil or stent assisted	391	1	95.4	yes
Radboud University Medical Center	Netherlands	simple coil	469	2	57.2	yes
Radcliffe Infirmary	United Kingdom	simple coil	327	1	86.1	yes
Radcliffe Infirmary	United Kingdom	simple coil	69	1	31.4	yes
Royal Melbourne Hospital	Australia	simple coil	371	1	27.1	yes
Royal Perth Hospital	Australia	simple coil	51	1	11.9	no
S. Raffaele Hospital	Italy	simple coil	105	2	10.5	no
Seoul National University Hospital	Korea	simple coil or stent assisted	449	1	47.3	yes
Seoul National University Hospital	Korea	simple coil	174	1	22.3	yes
Severance Hospital	Korea	simple coil	486	1	35.79	yes
Shanghai Sixth People’s Hospital	China	simple coil	221	1	31.6	yes
Soonchunhyang University Cheonan Hospital	Korea	simple coil or stent assisted	240	1	28.6	yes
St Elisabeth Ziekenhuis	Netherlands	simple coil or balloon assisted	681	1	64.2	yes
St. Elisabeth Hospital	Netherlands	simple coil	182	1	33.1	yes
Tampere University Hospital	Finland	simple coil or stent assisted	491	1	35.1	yes
Tampere University Hospital	Finland	simple coil	29	1	9.4	no

Tangdu Hospital, Fourth Military Medical University	China	simple coil or stent assisted	102	1	51	yes
TCM Hospital of Guangdong Province	China	simple coil	161	1	40.3	yes
Thomas Jefferson University Hospital	United States of America	simple coil or balloon assisted	29	1	16.1	no
Toronto Western Hospital	Canada	simple coil or balloon assisted	377	1	26.9	yes
University at Buffalo Neurosurgery	United States of America	simple coil or balloon assisted	187	1	46.8	yes
University Hospital Cologne	Germany	simple coil	236	1	23.8	yes
University Hospital Essen	Germany	simple coil or balloon assisted	85	1	10.5	no
University Medical Centre Utrecht	Netherlands	simple coil	160	1	32	yes
University of California at Los Angeles	United States of America	simple coil	403	8	10.7	no
University of Essen Medical School	Germany	simple coil	164	1	37.1	yes
University of Minnesota School of Medicine	United States of America	simple coil	525	3	29.2	yes
University of Pittsburgh Medical Center	United States of America	simple coil or balloon assisted	118	1	28.8	yes
University of Reims	France	simple coil or stent assisted	138	16	10.35	no
University of Torino	Italy	simple coil	448	1	37.3	yes
University of Toronto	Canada	simple coil or stent assisted	95	1	17.9	no
University of Turin	Italy	simple coil	378	1	23.6	yes
University of Washington	United States of America	simple coil	87	1	58	yes
Uppsala University Hospital	Sweden	simple coil	239	1	55.6	yes
Wakayama Medical University	Japan	simple coil	57	1	19	no
Yeungnam University	Korea	simple coil or balloon assisted	115	1	115	yes
Zaporizhzhya Regional Clinical Hospital	Ukraine	simple coil or stent assisted	247	1	22.5	yes
Zhujiang Hospital	China	simple coil	1308	1	186.9	yes

Supplement Material S7. Full forest plot of IPR among high- and low-volume centers

Study or Subgroup	Events	Total	Events per 100 observations GLMM, Random, 95% CI
**volume = high**			
Klinikum Stuttgart	2	452	0.44 [0.05; 1.59]
First Hospital Hebei Medical University	2	244	0.82 [0.10; 2.93]
CHU de Bicêtre	3	320	0.94 [0.19; 2.72]
Centre Hospitalier Universitaire Nice	2	195	1.03 [0.12; 3.66]
University of Washington	1	87	1.15 [0.03; 6.24]
Changhai Hospital	5	414	1.21 [0.39; 2.80]
University Medical Centre Utrecht	2	160	1.25 [0.15; 4.44]
Hopital Erasme	6	457	1.31 [0.48; 2.84]
Zhujiang Hospital	18	1308	1.38 [0.82; 2.17]
Neurosurgical Centre Nijmegen	4	285	1.40 [0.38; 3.55]
Beth Israel Hyman-Newman Institute	6	414	1.45 [0.53; 3.13]
Okayama University Medical School	2	134	1.49 [0.18; 5.29]
Guro Hospital	1	64	1.56 [0.04; 8.40]
Mito Medical Center	4	247	1.62 [0.44; 4.09]
Severance Hospital	8	486	1.65 [0.71; 3.22]
Yeungnam University	2	115	1.74 [0.21; 6.14]
University of Turin	7	378	1.85 [0.75; 3.78]
University of Minnesota School of Medicine	10	525	1.90 [0.92; 3.47]
Seoul National University Hospital	9	449	2.00 [0.92; 3.77]
Beth Israel Hyman-Newman Institute	1	48	2.08 [0.05; 11.07]
Seoul National University Hospital	4	174	2.30 [0.63; 5.78]
Changhai Hospital	12	520	2.31 [1.20; 4.00]
Clinical Center of Serbia	5	206	2.43 [0.79; 5.57]
Karadeniz Technical University Farabi Hospital	12	481	2.49 [1.30; 4.32]
Lariboisière Hospital	4	158	2.53 [0.69; 6.35]
Radboud University Medical Center	12	469	2.56 [1.33; 4.43]
Konyang University Hospital	5	185	2.70 [0.88; 6.19]
G B Pant Hospital	4	132	3.03 [0.83; 7.58]
University of Essen Medical School	5	164	3.05 [1.00; 6.97]
Tampere University Hospital	15	491	3.05 [1.72; 4.99]
Karol Marcinkowski Medical University	4	116	3.45 [0.95; 8.59]
First Affiliated Hospital of Dalian Medical University,	10	279	3.58 [1.73; 6.49]
Huashan Hospital	23	637	3.61 [2.30; 5.37]
Zaporizhzhya Regional Clinical Hospital	9	247	3.64 [1.68; 6.80]
Centre Hospitalier Universitaire Bretonnea	25	683	3.66 [2.38; 5.36]
Medical University of Bialystok	7	190	3.68 [1.49; 7.44]
ARETA	28	753	3.72 [2.48; 5.33]
First Affiliated Hospital of China Medical University	14	370	3.78 [2.08; 6.27]
St. Elisabeth Hospital	7	182	3.85 [1.56; 7.76]
Uppsala University Hospital	10	239	4.18 [2.02; 7.56]
University Hospital Cologne	10	236	4.24 [2.05; 7.65]
University of Torino	19	448	4.24 [2.57; 6.54]
CLARITY	34	782	4.35 [3.03; 6.02]
Radcliffe Infirmary	3	69	4.35 [0.91; 12.18]
Alfried Krupp Krankenhaus	45	1034	4.35 [3.19; 5.78]
Hospital Universitario Central de Asturias	11	251	4.38 [2.21; 7.71]
Hôpital Henri Mondor	4	91	4.40 [1.21; 10.87]
Shanghai Sixth People's Hospital	10	221	4.52 [2.19; 8.16]
Fondation Ophtalmologique Adolphe de Rothschild	6	132	4.55 [1.69; 9.63]
St Elisabeth Ziekenhuis	31	681	4.55 [3.11; 6.40]
Qilu Hospital of Shandong University	18	391	4.60 [2.75; 7.18]
Kyungpook National University Hospital	16	332	4.82 [2.78; 7.71]
Radcliffe Infirmary	17	327	5.20 [3.06; 8.19]
Frenchay Hospital	37	711	5.20 [3.69; 7.10]
New York Presbyterian Hospital	19	352	5.40 [3.28; 8.30]
Dr. Ram Manohar Lohia Institute of Medical Sciences	5	90	5.56 [1.83; 12.49]
Haukeland University Hospital	5	83	6.02 [1.98; 13.50]
Toronto Western Hospital	24	377	6.37 [4.12; 9.32]
Enfant-Jésus Hospital	34	532	6.39 [4.47; 8.82]
Gold Coast Hospital	2	30	6.67 [0.82; 22.07]
Royal Melbourne Hospital	26	371	7.01 [4.63; 10.10]
TCM Hospital of Guangdong Province	12	161	7.45 [3.91; 12.66]
University at Buffalo Neurosurgery	14	187	7.49 [4.15; 12.24]
University of Pittsburgh Medical Center	9	118	7.63 [3.55; 13.99]
Hôpital Purpan, University of Toulouse	17	222	7.66 [4.52; 11.98]
Kuopio University Hospital	4	52	7.69 [2.14; 18.54]
Kurashiki Central Hospital	9	117	7.69 [3.58; 14.10]
Massachusetts General Hospital	12	156	7.69 [4.04; 13.05]
Azienda Ospedaliero-Universitaria Policlinico Umberto I	17	215	7.91 [4.67; 12.36]
Centre Hospitalier de l'Universite de Montreal	6	75	8.00 [2.99; 16.60]
Tangdu Hospital, Fourth Military Medical University	9	102	8.82 [4.11; 16.09]
Careggi University Hospital	55	510	10.78 [8.23; 13.81]
Soonchunhyang University Cheonan Hospital	28	240	11.67 [7.89; 16.42]
**Total (95% CI)**		**23154**	**3.50 [3.01; 4.06]**
Heterogeneity: $Tau^2 = 0.3133$; $Chi^2 = 281.39$, df = 72 ($P < 0.01$); $I^2 = 74\%$			
**volume = low**			
Mayo Clinic	1	83	1.20 [0.03; 6.53]
Hospital Clínico San Carlos	1	66	1.52 [0.04; 8.16]
ISAT-2	1	48	2.08 [0.05; 11.07]
Kyoto Prefectural Yosanoumi Hospital	1	38	2.63 [0.07; 13.81]
University of California at Los Angeles	11	403	2.73 [1.37; 4.83]
S. Raffaele Hospital,	3	105	2.86 [0.59; 8.12]
University of Reims	4	138	2.90 [0.80; 7.26]
Tampere University Hospital	1	29	3.45 [0.09; 17.76]
Thomas Jefferson University Hospital	1	29	3.45 [0.09; 17.76]
Wakayama Medical University	2	57	3.51 [0.43; 12.11]
CHR de la Citadelle	1	26	3.85 [0.10; 19.64]
JR-NET	227	5102	4.45 [3.90; 5.05]
University Hospital Essen	4	85	4.71 [1.30; 11.61]
GEL THE NEC	10	194	5.15 [2.50; 9.28]
CARAT	16	299	5.35 [3.09; 8.54]
ISAT	9	141	6.38 [2.96; 11.77]
Oregon Health Sciences University	2	31	6.45 [0.79; 21.42]
Hanyang University Medical Center	6	80	7.50 [2.80; 15.61]
Prince of Wales Hospital	6	80	7.50 [2.80; 15.61]
University of Toronto	8	95	8.42 [3.71; 15.92]
Medical University of Silesia	7	77	9.09 [3.73; 17.84]
Millard Fillmore Hospital	6	58	10.34 [3.89; 21.17]
Royal Perth Hospital	7	51	13.73 [5.70; 26.26]
Kokura Memorial Hospital	4	28	14.29 [4.03; 32.67]
Ludwig-Maximilians-University	20	93	21.51 [13.66; 31.24]
**Total (95% CI)**		**7436**	**5.37 [4.06; 7.07]**
Heterogeneity: $Tau^2 = 0.2926$; $Chi^2 = 80.33$, df = 24 ($P < 0.01$); $I^2 = 70\%$			
**Total (95% CI)**		**30590**	**3.81 [3.32; 4.37]**
Heterogeneity: $Tau^2 = 0.3434$; $Chi^2 = 367.70$, df = 97 ($P < 0.01$); $I^2 = 74\%$			
Test for subgroup differences: $Chi^2 = 6.99$, df = 1 ($P < 0.01$)			

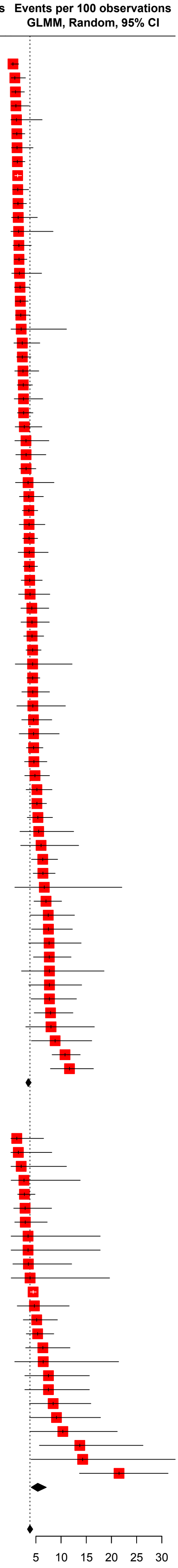

Supplement: Supplementary file 1 [file life-16-01393-s001.zip › life-4449241-supplementary.pdf]
